# Ecological Succession of Airborne Bacterial Aerosols in Poultry Houses: Insights from Taihang Chickens

**DOI:** 10.3390/ani15243635

**Published:** 2025-12-17

**Authors:** Yejin Yang, Huan Cui, Zitong Yang, Zhenyue Li, Wenhao Feng, Zhuhua Liu, Mengxi Yan, Zhibin Ren, Ran Zhu, Yuqing Yang, Mingli Liu, Xiaolong Chen, Cheng Zhang, Huage Liu, Shishan Dong

**Affiliations:** 1 College of Veterinary Medicine, Hebei Agricultural University, Baoding 071000, China; 2 The Animal Husbandry and Veterinary Institute of Hebei, Baoding 071001, China; 3 College of Medicine, Yanbian University, Yanji 133002, China; 4 College of Animal Science and Technology, Hebei North University, Zhangjiakou 075000, China

**Keywords:** bioaerosols, particle size distribution, bacterial community, Taihang chicken, intensive poultry housing

## Abstract

Bioaerosols are a major source of airborne microbial contamination in intensive poultry production systems. Their concentration and community structure can profoundly influence animal health, public health, and the overall safety of the farming environment. However, the dynamic characteristics of bacterial aerosols in enclosed poultry houses during winter remain insufficiently studied. Using Taihang chickens as a model, this study investigated three key production stages—brooding (15 days), growing (60 days), and laying (150 days)—under winter cage-rearing conditions. A six-stage Andersen sampler was employed alongside culture-dependent enumeration and 16S rRNA high-throughput sequencing to analyze variations in bacterial aerosol concentration, particle size distribution, and community succession patterns. The results revealed a significant increase in the concentration of culturable airborne bacteria with bird age, rising from 8.98 × 10^3^ colony-forming unit (CFU)/m^3^ to 2.89 × 10^4^ CFU/m^3^ (*p* < 0.001). The particle size distribution progressively shifted from larger, settleable particles (≥4.7 μm) toward smaller, respirable particles (<4.7 μm). Microbial sequencing indicated a continuous increase in bacterial alpha diversity across the three stages (Chao1 and Shannon indices, *p* < 0.05), while beta diversity exhibited stage-specific clustering, reflecting clear differences in community assembly. The composition of dominant bacterial genera transitioned from potentially pathogenic taxa such as *Acinetobacter* and *Corynebacterium* during the brooding stage to a greater abundance of beneficial genera, including *Bacteroides*, *Lactobacillus*, and *Ruminococcus*, in later stages. This shift suggests a potential ecological link between aerosolized bacterial communities and host development, possibly related to the aerosolization of gut microbiota. Notably, several zoonotic bacterial species were detected in the poultry house air, indicating potential public health and occupational exposure risks under winter confinement conditions. This study is the first to elucidate the ecological succession patterns of airborne bacterial aerosols in Taihang chicken houses across different growth stages during winter. The findings provide a scientific basis for optimizing winter ventilation strategies, implementing stage-specific environmental controls, and reducing pathogen transmission and occupational hazards.

## Simple Summary

Understanding the air quality inside modern chicken houses is essential for protecting both animal health and the well-being of farm workers. In winter, poultry farms often reduce ventilation to maintain warmth, which unintentionally allows airborne bacteria to build up. Our study examined the air inside Taihang chicken houses during three important stages of growth—early chicks, growing birds, and adult layers—under typical winter conditions. We found that the amount of airborne bacteria increased steadily as the birds grew and that smaller particles capable of reaching deeper into the lungs became more common over time. We also discovered that the types of bacteria in the air changed with age, shifting from environmental and potentially harmful species in young-chick environments toward more diverse communities that included bacteria originating from the birds’ own digestive systems. These findings show that winter creates unique challenges for air quality management in poultry houses and highlight when bacterial exposure risks are likely to be highest. Our results provide valuable guidance for improving ventilation, hygiene, and the working environment on farms, ultimately supporting healthier flocks, safer workplaces, and more sustainable poultry production.

## 1. Introduction

As one of the world’s largest producers and consumers of poultry, China remains a global leader in both poultry meat and egg production. According to the latest FAO livestock and poultry outlook report, national poultry meat output exceeded 24 million tons in 2023, and egg production surpassed 35 million tons, ranking first worldwide [1]. This sector plays a vital role in ensuring national food security and supplying high-quality dietary protein. With the ongoing intensification and modernization of animal husbandry, enclosed cage systems have been widely implemented in cold northern regions to improve production efficiency, facilitate controlled waste management, and enhance environmental regulation [2,3]. However, such confined and high-density rearing systems also promote the accumulation of bioaerosols within poultry houses. These aerial pollutants comprise a complex mixture of particulate matter, culturable and unculturable microorganisms—including bacteria, fungi, and viruses—as well as endotoxins and noxious gases such as ammonia and hydrogen sulfide [4,5]. These components pose potential threats to animal welfare, the respiratory health of farm workers, and the surrounding environment [6,7,8].

Previous studies have established that inhalable fine particulate matter, particularly particles in the particulate matter (PM) 2.5–PM4.7 size range, can serve as carriers for bacterial aerosols. Experimental and occupational exposure studies have demonstrated that such particles induce oxidative stress responses and alter respiratory immune function, including airway inflammation and immune dysregulation [8,9]. Workers subjected to long-term exposure to high aerosol concentrations exhibit increased incidence of airway hypersensitivity, chronic obstructive pulmonary disease, and respiratory infections [7]. Moreover, airborne transmission of pathogenic or zoonotic microorganisms within poultry houses may not only accelerate intra-flock disease spread but also raise public health concerns for both farm workers and nearby communities [6,8,10].

Winter conditions exert a particularly significant influence on this issue. In northern China, low ambient temperatures often lead to reduced ventilation in poultry houses as a means of conserving heat. While this strategy lowers energy consumption, it also encourages the buildup of particulate matter, microorganisms, and harmful gases, thereby modifying their transport behavior, size distribution, and biological load [9,10]. The balance between ventilation and insulation creates distinct environmental selection pressures that shape the structure of microbial aerosol communities. Studies have indicated that the microbial community in poultry houses during winter differs markedly from that in other seasons, typically showing higher microbial density, greater complexity in microbial networks, and an elevated prevalence of potential pathogens [6]. Nevertheless, current understanding of the ecological dynamics of airborne bacterial aerosols in enclosed poultry environments—particularly under winter minimum-ventilation conditions and across different growth stages—remains limited, as existing studies have primarily focused on overall microbial burden rather than stage-resolved community succession [9,11]. Whether aerosol composition follows stage-specific patterns is still an open question.

The Taihang chicken, a native breed from Hebei Province, is valued for its dual-purpose meat and egg production, adaptability to low-quality feed, strong disease resistance, superior meat quality, and well-developed immune system [12,13]. As consumer demand for high-quality and specialty animal products continues to grow, the Taihang chicken industry has expanded substantially. Taihang chickens are known for their strong adaptability, slow-growing characteristics, and high-quality eggs. Recent statistics indicate that the national flock size has reached approximately 20 million birds, producing more than 190,000 tons of high-quality eggs annually [14]. Typical production data show that Taihang hens produce 165–185 eggs per bird per year, with an average daily feed intake of 95–110 g and a feed conversion ratio of 2.5–2.8 during the laying period [15]. These production traits highlight the economic relevance of this breed and underscore the importance of optimizing its housing environment. Accompanying this growth is a rapid shift from traditional free-range systems to high-density, intensive, and facilities-based production models [16]. Under winter enclosed-cage conditions, notable differences in feed intake, activity levels, respiratory excretion, and metabolic output occur across developmental stages—such as the brooding, growing, and laying periods [17,18,19]. These variations are likely to drive stage-dependent shifts in the concentration, size distribution, dominant species, and pathogen-related risks of bacterial aerosols [20]. Without effective control of aerosol pollution during winter, even excellent genetic stock, high-quality feed, and strict vaccination protocols may fail to ensure optimal production performance [21].

Although recent research has addressed seasonal variation, exposure risks, and mitigation strategies related to bioaerosols in livestock buildings, systematic investigations targeting the multi-factor interplay among winter enclosed-cage environments, local poultry breeds, different production stages, and bacterial aerosol ecological succession are still lacking [22,23]. Therefore, this study conducted systematic monitoring of bacterial aerosols in the air of typical enclosed Taihang chicken houses in Hebei Province during winter, covering the brooding, growing, and laying periods. Using a six-stage Andersen sampler coupled with 16S rRNA high-throughput sequencing, we aimed to characterize the temporal dynamics of aerosol concentration, particle size distribution, biodiversity, and community structure throughout the winter period. Our findings are expected to advance the understanding of microbial aerobiology in enclosed poultry environments under winter conditions. Importantly, the stage-specific patterns identified in this study can be incorporated into practical management guidelines by informing winter ventilation adjustments, refining stage-dependent cleaning and disinfection schedules, and supporting targeted monitoring of airborne microbial loads. These applications provide a scientific basis for improving flock productivity and reducing occupational and public health risks in cold-region poultry systems. This study provides the first stage-resolved characterization of bacterial aerosol dynamics in winter poultry houses, offering new insights into how restricted ventilation and host developmental factors jointly shape airborne microbiomes.

## 2. Materials and Methods

### 2.1. Site Description and Experimental Design

This study was conducted during the winter (December 2024) at five standardized, large-scale cage-layer farms housing Taihang chickens in Hebei Province. All poultry houses were fully enclosed with a multi-tiered staircase cage design, equipped with mechanical ventilation and centralized manure-belt cleaning systems. Each poultry house contained approximately 12,000–15,000 Taihang chickens housed in a fully enclosed. Each cage housed 5 birds, corresponding to a stocking density of approximately 4.17 birds/m^2^. Manure was removed by the automatic belt system every 48 h. During the winter season, the houses operated under routine thermal insulation and a minimum-ventilation mode, maintaining an air speed of ≤0.20 m/s. To characterize the dynamics of airborne bacterial aerosols across different growth stages, sampling was conducted at three representative phases of the production cycle: the brooding stage at approximately 1–3 weeks of age (15 days), the growing stage at approximately 8–10 weeks of age (60 days), and the early laying stage at approximately 20–23 weeks of age (150 days). Taihang chickens typically reach the onset of lay at approximately 140–160 days of age (20–23 weeks), with an average body weight of 1.35–1.55 kg at first lay. The 150-day sampling point in this study therefore corresponds to the early laying stage, when reproductive maturity and metabolic demand increase substantially. The environmental conditions within the houses were maintained as follows: Brooding stage (15 days): 30–35 °C, 60–70% relative humidity; houses operated under minimum winter ventilation with an air speed ≤ 0.20 m/s. Growing stage (60 days): 22–27 °C, 55–65% relative humidity; ventilation and heating adjusted to maintain stable airflow and thermal insulation. Laying stage (150 days): 15–20 °C, 50–60% relative humidity; minimum-ventilation mode maintained with controlled airflow to prevent heat loss. These controlled environmental parameters reflect typical winter operational conditions in enclosed multi-tier cage systems. Air sampling at 15, 60, and 150 days was conducted using a cross-sectional design across different flocks housed in the same type of enclosed Taihang chicken houses. All houses had identical structural design, stocking density standards, cage configuration, and ventilation settings. Each flock was sampled at the corresponding production stage, following the same management schedule. This design ensured that differences observed among stages reflected biological and production-stage characteristics rather than variations in housing or density.

A total of five fixed sampling points were established within each house at each farm and for each growth stage. These points were strategically located in ventilation zones at the front, middle, and rear of the house, as well as at upper and lower tiers representing typical breathing heights. All sampling at these five points within a single house was completed on the same day. This design resulted in a total of 75 air samples, calculated as: 5 farms × 3 growth stages × 5 sampling points = 75 samples. The five samples collected per stage were assigned to two analytical approaches. For culturable bacterial counting, all samples were analyzed individually to allow statistical comparison of airborne bacterial load and particle size distribution. For 16S rRNA gene sequencing, however, the five samples were pooled into one composite sample. This pooling strategy is commonly used in bioaerosol studies to obtain sufficient microbial biomass for reliable DNA extraction, particularly under winter minimum-ventilation conditions, and to generate a representative profile of the overall airborne bacterial community at each growth stage.

16S rRNA High-Throughput Sequencing Analysis: To profile the overall composition and diversity of the airborne bacterial community, the biomass from the five samples obtained from the same farm and stage was pooled into a single composite sample. This pooling strategy yielded a total of 15 composite samples for sequencing (5 farms × 3 stages).

This integrated sampling strategy ensured statistical robustness for quantifying culturable microbial load while maintaining representativeness and cost-effectiveness for community structure analysis, adhering to common experimental design principles in poultry aerosol research.

### 2.2. Airborne Microorganism Sampling and Culturable Counting

The six-stage impactor, operating at a flow rate of 28.3 L·min^−1^, fractionated particles according to the following aerodynamic diameters: Stage 1: ≥7.0 μm; Stage 2: 4.7–7.0 μm; Stage 3: 3.3–4.7 μm; Stage 4: 2.1–3.3 μm; Stage 5: 1.1–2.1 μm; and Stage 6: 0.65–1.1 μm. At each sampling point, collection was conducted at a height of approximately 1.2–1.5 m above the ground, corresponding to the typical breathing zone of the chickens. A single sampling volume of 300 L (over a duration of 10.6 min) was collected per point. Each stage of the impactor was loaded with a pre-poured Tryptic Soy Agar (TSA) plate.

Following sampling, the plates were incubated at 37 °C for 24–48 h for colony enumeration. To monitor potential contamination, both transport blanks (unopened plates carried to and from the site) and field exposure blanks (plates opened at the site without active sampling) were included during each sampling event. The concentration of culturable bacteria in CFU per cubic meter of air (CFU·m^−3^) was calculated according to established methods [10].

### 2.3. Aerosol Sample Processing and DNA Extraction

The airborne microorganisms were collected following a method adapted from previous studies. Aerosol sampling was conducted using a high-volume air sampler (Model HH02-LS120, Beijing Hvarian Nuclear Safety Technology Co., Ltd., Beijing, China) loaded with pre-sterilized Tissuquartz™ quartz fiber filters (20.32 cm × 25.4 cm, PALL, New York, NY, USA). To ensure collection efficiency and sample representativeness, the sampler operated continuously for 12 h at a high flow rate of 1000 L/min [24]. Sampling was simultaneously performed at five designated points within the poultry house of each of the five Taihang chicken farms during the brooding, growing, and laying stages. After the 12-h collection, the filters from these five parallel sampling points were pooled in the laboratory to form one composite sample per house per stage for subsequent nucleic acid analysis. To minimize background contamination, all filters were pre-treated by baking in a muffle furnace at 500 °C for 48 h. Post-sampling, each filter was immediately placed into a sterile zip-lock bag, transported to the laboratory in a portable cooler, and stored at −80 °C until processing. Prior to DNA extraction, each filter was cut into small pieces of approximately equal weight (weight difference ≤ 1 mg) using sterile scissors. The pieces were transferred to a sterile 50 mL centrifuge tube, and ultrapure water was added, followed by vigorous vortexing to release microorganisms from the filter into the aqueous phase. The resulting suspension was centrifuged at 25,000× *g* for 10 min at 4 °C to pellet the total biomass of bioaerosol particles. Total genomic DNA was then extracted from the pellet using the cetyltrimethylammonium bromide (CTAB) method. The concentration and purity of the extracted DNA were measured using a NanoDrop 2000 spectrophotometer (Thermo Fisher Scientific, Waltham, MA, USA), and its integrity was assessed by 0.7% agarose gel electrophoresis. For samples that failed to meet the quality standards, DNA re-extraction was performed until the quality was sufficient for downstream amplification and library construction.

### 2.4. 16S rRNA Amplicon Sequencing and Library Construction

The V3–V4 hypervariable region of the bacterial 16S rRNA gene was amplified using the primer pair 341F (5′-CCTACGGGNGGCWGCAG-3′) and 806R (5′-GGACTACHVGGGTWTCTAAT-3′). The polymerase chain reaction (PCR) was performed in a total volume of 25 μL. The reaction mixture comprised 12.5 μL of 2× high-fidelity master mix, 0.2 μM of each primer, and 10–20 ng of template DNA. The thermal cycling protocol consisted of an initial denaturation at 95 °C for 3 min; followed by 30–32 cycles of denaturation at 95 °C for 30 s, annealing at 55 °C for 30 s, and extension at 72 °C for 45 s; with a final extension at 72 °C for 5 min. Negative and positive controls were included in each amplification batch. The PCR products were verified for quality and specificity via 2% agarose gel electrophoresis and subsequently purified using a gel extraction kit (Qiagen, Hilden, Germany). The purified amplicons were pooled in equimolar amounts for library construction. Sequencing libraries were prepared using the TruSeq DNA PCR-Free Sample Preparation Kit (Illumina, San Diego, CA, USA) and sequenced on an Illumina HiSeq 2500 platform for 250 bp paired-end reads. Primary quality control of the raw sequencing data and data delivery were performed by Novogene Co., Ltd. (Beijing, China). The sequencing data have been deposited in the NCBI GenBank database under BioProject accession number PRJNA1354577.

### 2.5. Bioinformatic Analysis of 16S rRNA Gene Sequencing Data

Raw sequencing reads were first processed for quality control using QIIME2 (version 2025.4). This step involved the removal of adapter contaminants, low-quality sequences, and ambiguous nucleotides to generate high-quality clean tags. Subsequently, chimeric sequences were detected and filtered out using UCHIME (version 4.2). The resulting effective tags were then clustered into Operational Taxonomic Units (OTUs) at a 97% similarity threshold using Uparse (version 7.0.1001), from which representative sequences for each OTU were selected. Taxonomic annotation of these representative sequences was performed against the SILVA 132 database to obtain phylogenetic information at levels from phylum to genus. For community diversity analysis, alpha diversity, reflecting microbial richness and diversity within samples, was assessed using the Chao1 and Shannon indices. Beta diversity, which measures compositional differences between samples, was calculated using Bray–Curtis dissimilarity based on OTU profiles and visualized through Principal Coordinates Analysis (PCA) to reveal structural similarities and differences among microbial communities. Statistically significant differentially abundant taxa were identified through comparative analysis, and their distribution patterns were visualized using clustered heatmaps and stacked bar plots to illustrate the overall differences in airborne bacterial community composition across the various growth stages.

### 2.6. Statistical Analysis

Statistical analyses of airborne bacterial concentrations and diversity indices were performed using SPSS (version 19.0, SPSS Inc., Chicago, IL, USA) and GraphPad Prism (version 8.0, GraphPad Software, San Diego, CA, USA). Differences across growth stages were assessed using one-way analysis of variance (one-way ANOVA). Post-hoc pairwise comparisons were conducted using Tukey’s Honestly Significant Difference (HSD) test to control for multiple testing, with statistical significance set at *p* < 0.05. All experimental data are presented as mean ± standard deviation (mean ± SD).

## 3. Results

### 3.1. Concentration and Size Distribution of Airborne Culturable Bacteria

**Figure 1:**
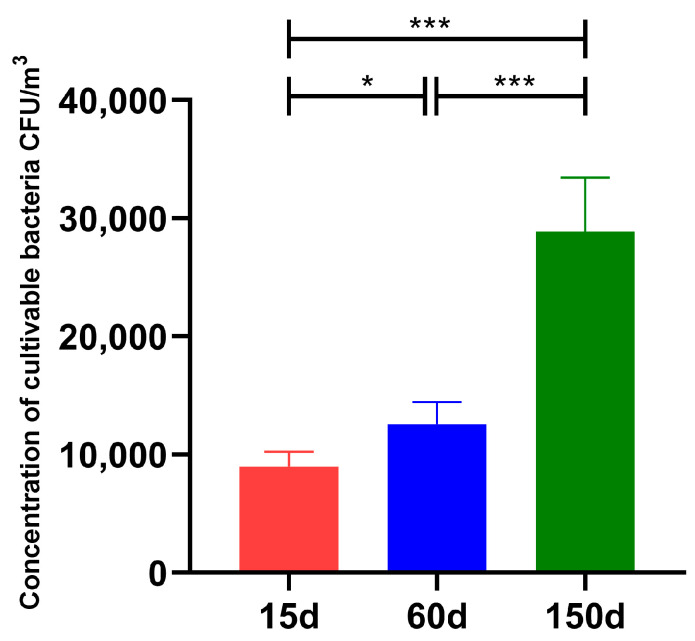
Dynamics of culturable bacterial concentrations in the air of enclosed Taihang chicken houses during three growth stages in winter (*n* = 5 farms × 5 sampling points). Bacterial concentrations at 15, 60, and 150 days of age progressively increased, ranging from 10^3^ to 10^4^ CFU/m^3^ (mean ± SD), with statistically significant differences between stages (* *p* < 0.05; *** *p* < 0.001).

The concentration of airborne culturable bacteria demonstrated significant dynamic changes across different growth stages of the Taihang chickens (Figure 1). At 15 days of age, the bacterial concentration was 8.98 × 10^3^ ± 1.26 × 10^3^ CFU/m^3^. A significant increase to 1.26 × 10^4^ ± 1.86 × 10^3^ CFU/m^3^ was observed at 60 days of age (*p* < 0.05). The concentration further rose markedly to 2.89 × 10^4^ ± 4.58 × 10^3^ CFU/m^3^ by 150 days of age (*p* < 0.001). Notably, the concentration at 150 days was also significantly higher than that at 60 days (*p* < 0.001). This progressive accumulation of bacterial bioaerosols with increasing flock age and metabolic activity is likely associated with enhanced microbial emissions from the chickens, manure fermentation, and limited ventilation efficiency in the poultry houses.

### 3.2. Size Distribution of Culturable Bacteria

**Figure 2:**
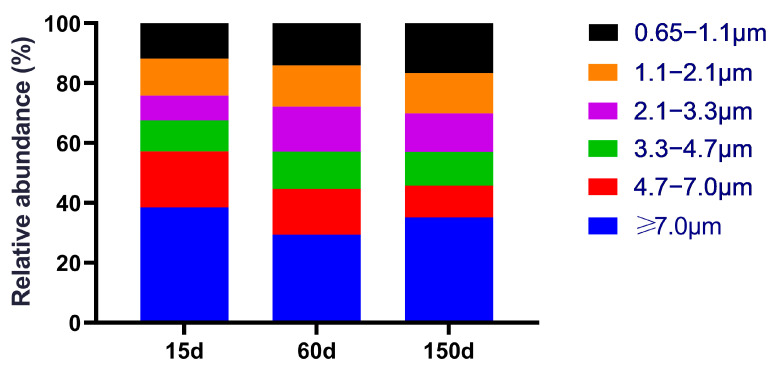
Size-fractionated distribution of culturable bacteria across three growth stages (15, 60, and 150 days). The distribution shifted from large, settleable particles (≥4.7 μm) dominating at 15 days toward significantly increased proportions of respirable particles (<4.7 μm) at 60 and 150 days, accompanied by notable enrichment in the 3.3–4.7 μm size range. *n* = 5 farms × 5 sampling points.

The size distribution of culturable bacteria also exhibited significant stage-specific dynamics (Figure 2). At 15 days of age, bacteria were predominantly associated with large particles (≥4.7 μm, 57.18%), with the highest proportion found in the ≥7.0 μm fraction (38.56%). This pattern suggests that the airborne bacteria during the early stage primarily originated from settleable particulates, such as fecal dust, feather debris, and feed fragments. By 60 days of age, the proportion of large particles decreased to 44.65%, while the fraction in the 3.3–4.7 μm range peaked at 12.46%. This shift indicates a transition towards respirable particle sizes capable of depositing in the deeper respiratory tract. A fine-particle-dominant profile emerged at 150 days of age, with small particles (<4.7 μm) constituting 54.21% of the total, despite a slight rebound in large particles (45.79%). Given that fine particles possess prolonged airborne residence times, higher respiratory deposition efficiency, and greater potential for inducing inflammation, this particle-size transition signifies a dual increase in airborne exposure risk—both in quantity and potential toxicity—as the flock ages during winter. This trend not only elevates the risk of oxidative stress in poultry but also raises potential health concerns for farm workers due to increased exposure.

### 3.3. General Sequencing Characteristics and OTUs Abundance

**Figure 3:**
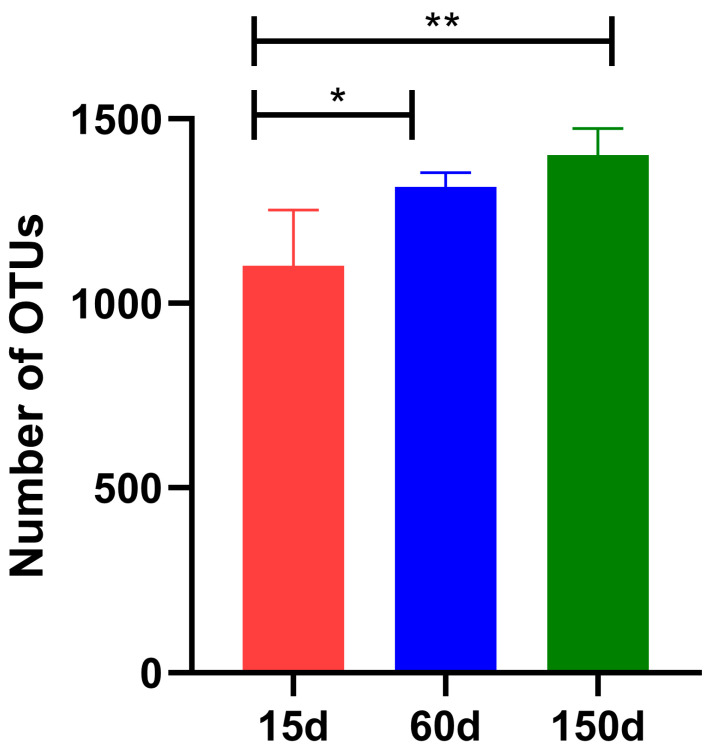
Comparison of Operational Taxonomic Units (OTUs) richness in air samples across different growth stages (*n* = 5 farms × 5 sampling points). OTU counts were lowest at 15 days, increased significantly at 60 days, and further elevated at 150 days (* *p* < 0.05; ** *p* < 0.01).

After quality control, a total of 5.97 × 10^4^ high-quality effective sequences were obtained. Rarefaction curves for all samples approached a plateau, indicating adequate sequencing depth for diversity analysis (Supplementary Figure S1). The OTUs demonstrated a significant increasing trend with flock age (Figure 3): 1.10 × 10^3^ ± 1.36 × 10^2^ at 15 days, rising to 1.32 × 10^3^ ± 0.38 × 10^2^ at 60 days (*p* < 0.05), and further increasing to 1.40 × 10^3^ ± 0.73 × 10^2^ at 150 days (*p* < 0.01). The rarefaction curves for all samples approached a plateau, indicating sufficient sequencing depth and confirming that the data were representative and reliable for subsequent analysis. These results reveal that within the enclosed environment during winter, the airborne bacterial community becomes progressively richer, concomitant with the physiological development and metabolic changes of the chickens.

### 3.4. Changes in Bacterial Community Alpha and Beta Diversity

**Figure 4:**
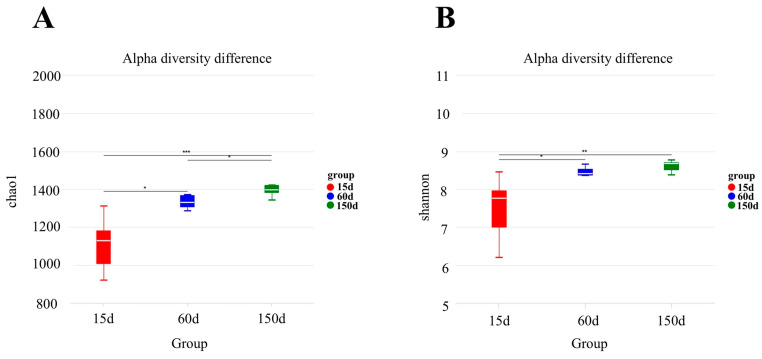
Changes in alpha diversity indices (Chao1 and Shannon) across 15, 60, and 150 days of age (*n* = 5 farms × 5 sampling points). The Chao1 index progressively increased (**A**). The Shannon index showed the most significant increase from 15 to 60 days (**B**). * *p* < 0.05, ** *p* < 0.01, *** *p* < 0.001.

Both the Chao1 and Shannon indices indicated a significant increase in the alpha diversity of the bacterial communities with increasing flock age (Figure 4). The Chao1 index rose from 1.11 × 10^3^ ± 1.50 × 10^2^ at 15 days to 1.33 × 10^3^ ± 3.90 × 10^1^ at 60 days, and further to 1.41 × 10^3^ ± 7.40 × 10^1^ at 150 days, with all pairwise comparisons being statistically significant (*p* < 0.05). The most pronounced increase in the Shannon index occurred between 15 days (7.50 ± 0.90) and 60 days (8.50 ± 0.10) (*p* < 0.05), followed by a more modest rise to 8.60 ± 0.20 at 150 days. These trends suggest that the richness and evenness of the airborne bacterial community were rapidly established during the mid-growth stage and subsequently stabilized.

**Figure 5:**
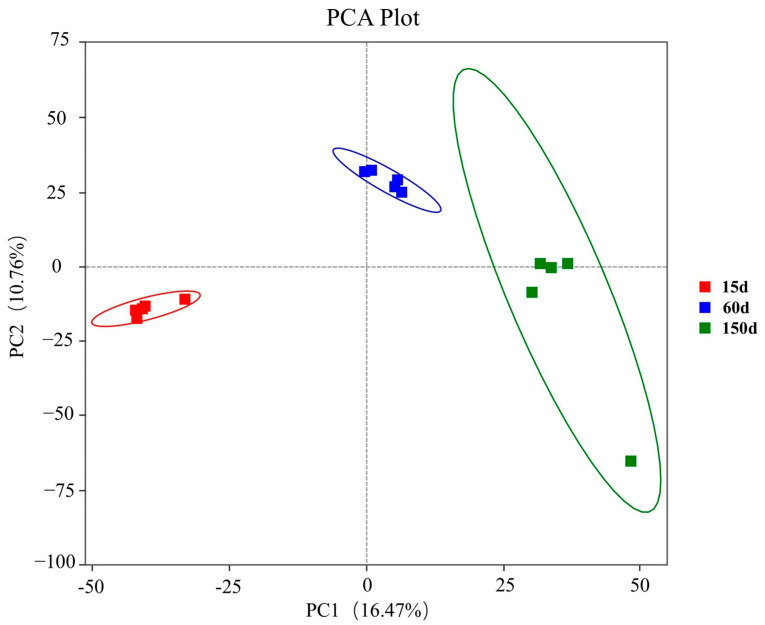
Principal Coordinates Analysis (PCA) of bacterial community β-diversity across the three growth stages based on Bray–Curtis dissimilarity (*n* = 5 farms × 5 sampling points).

PCA revealed a clear separation of bacterial community structures among the three growth stages (Figure 5). The distinct clustering of samples from each stage, with no overlap observed, indicates a time-dependent assembly pattern of the airborne bacterial communities in the Taihang chicken houses during winter. This structural divergence is likely driven by a combination of factors, including increased manure excretion, changes in stocking density, and the specific microclimate and ventilation strategies within the houses, which collectively exert selective pressures on the microbiota.

### 3.5. Bacterial Community Composition and Analysis of Potential Pathogens

**Figure 6:**
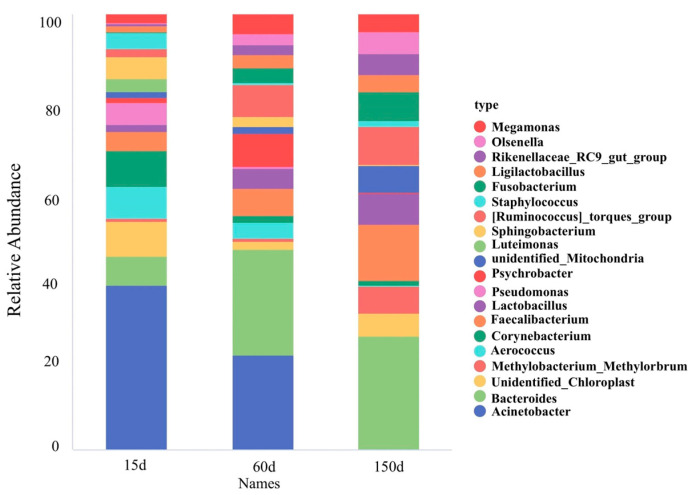
Ecological succession of dominant bacterial genera across three growth stages (*n* = 5 farms × 5 sampling points).

**Figure 7:**
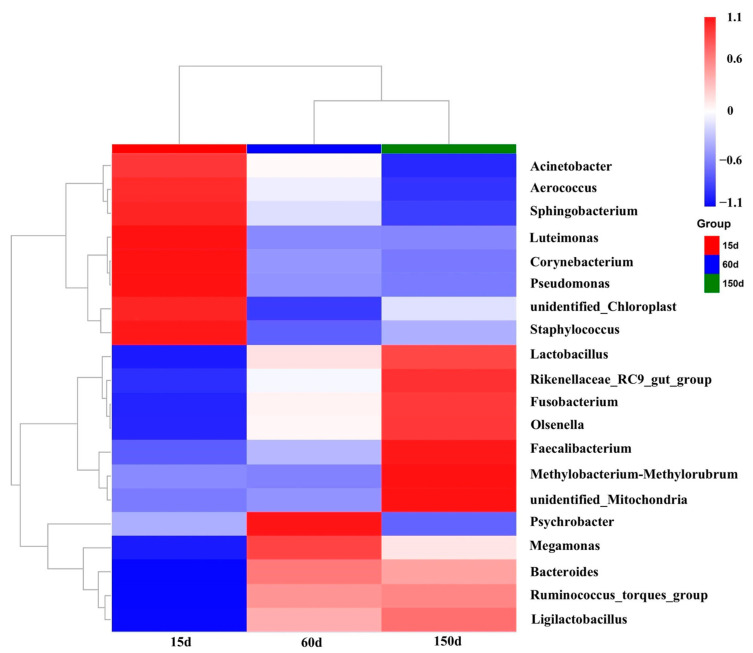
Heatmap clustering of the top 20 most abundant bacterial genera (*n* = 5 farms × 5 sampling points).

The composition of dominant bacterial genera demonstrated significant succession across the growth stages (Figure 6 and Figure 7). Beneficial or conditionally beneficial genera, such as *Bacteroides*, *Lactobacillus*, *Ruminococcus*, and *Faecalibacterium*, became increasingly predominant from 60 days of age onward. This shift reflects a progression towards a more complex and diversified community structure. However, several genera listed as zoonotic pathogens in national catalogs, including *Acinetobacter*, *Corynebacterium*, *Pseudomonas*, *Staphylococcus*, and *Fusobacterium*, were also detected in the winter housing environment. Notably, *Acinetobacter* accounted for a substantial 37.90% of the relative abundance at 15 days but declined rapidly thereafter. To provide quantitative support for the observed trends, detailed relative abundance values of dominant genera and families across the three growth stages are presented in Supplementary Table S1. This pattern suggests that early-stage airborne contamination was primarily dominated by environmental bacteria, whereas the increased presence of host-associated taxa in later stages may reflect greater biological activity and organic matter accumulation as birds mature.

## 4. Discussion

Airborne bioaerosols represent one of the most epidemiologically significant vectors of contamination in intensive poultry operations. They can carry bacteria, viruses, fungal spores, antibiotic resistance genes, and endotoxins, often coexisting with inhalable particulate matter and irritant gases such as ammonia [6]. This complex mixture poses considerable threats to poultry health, the respiratory systems of farm workers, and environmental safety [8]. Our study systematically elucidates the dynamics of bacterial aerosols in enclosed, cage-housed Taihang chicken houses during winter, characterizing the stage-dependent patterns of concentration accumulation, particle size migration, and community succession. From a microbial ecology perspective, our findings illustrate a dynamic process best described as “enclosure in winter → restricted ventilation → particulate accumulation → microbial succession → risk superposition.” Understanding this cascade is of immediate practical importance for implementing precise environmental control in poultry facilities located in cold regions [9,25]. By revealing the temporal succession of airborne bacterial communities across growth stages, this study advances the current understanding of winter poultry-house microbiology and identifies critical intervention windows for bioaerosol control.

This study demonstrated that the concentration of culturable airborne bacteria in Taihang chicken houses increased markedly across growth stages during winter, rising from 8.98 × 10^3^ CFU/m^3^ at 15 days to 2.89 × 10^4^ CFU/m^3^ at 150 days. Although our study did not directly quantify environmental drivers such as stocking density, manure accumulation, feather debris, or feed dust, the observed trend is consistent with patterns reported in previous poultry-house studies in which these factors have been proposed to contribute to elevated airborne microbial loads [9]. Moreover, low ventilation rates and dry winter conditions likely facilitate prolonged particle suspension and enhanced bacterial persistence [26]. This phenomenon aligns with earlier findings from livestock buildings in northern winter climates, suggesting that the winter environment itself has an amplifying effect on bacterial aerosol accumulation.

Of greater concern is the observed temporal shift in particle size distribution. As the flock aged, the aerosol profile transitioned from being dominated by large particles (which primarily deposit in the upper respiratory tract) to a finer-particle-dominated regime. These smaller particles can penetrate deeper into the lower respiratory tract and even reach the alveolar regions [27,28,29]. Particle size is a critical determinant of the deposition site within the host, the efficiency of pathogen carriage, airborne residence time, and the potential for cross-host transmission [30]. Consequently, the combination of “decreasing particle size + increasing concentration” signifies a dual escalation of airborne exposure risk during the later winter period [31]. This synergistic effect can amplify oxidative stress, immune suppression, and respiratory inflammation in poultry, while concurrently elevating the chronic occupational exposure risk for farm workers [32,33,34].

The microbial diversity analysis further confirmed a continuous succession of the aerosolized bacterial communities with flock age. The significant increase in alpha diversity, coupled with the clear separation of communities in the PCA plot for each stage, indicates that the structural shift was not a simple accumulation but an ecological transition from the dominance of environmental bacteria to host-associated taxa. The stage-specific restructuring of OTUs in the enclosed winter environment reflects a community reassembly mechanism driven by host development, immune maturation, manure accumulation, and indoor microclimate parameters. This dynamic aligns with the recently proposed concept of the “aerosolization of gut microbiota.”

In this study, several genera identified in the airborne bacterial community are recognized etiological agents of serious infections in both humans and animals. For instance, *Acinetobacter* can cause pneumonia and septicemia [35]; *Fusobacterium* is associated with Lemierre’s syndrome [36,37]; Corynebacterium can lead to respiratory inflammation and diphtheria [38]; and Pseudomonas and Staphylococcus are known to cause pyogenic infections in multiple organs [7,39]. Consequently, the enclosed poultry housing environment during winter presents a potential public health risk, warranting attention from both occupational health and environmental management perspectives. Our findings revealed that the aerosolized community exhibits a dual ecological character, co-harboring both potentially hazardous environmental pathogens and beneficial gut microbes closely linked to host health. On one hand, conditional pathogens such as *Acinetobacter*, *Corynebacterium*, *Pseudomonas*, *Staphylococcus*, and *Fusobacterium* were present at relatively high abundances during the early stage. This is likely attributable to the underdeveloped immune system of chicks, high environmental hygiene pressure, and frequent dispersal of excreta [11,24,26,40,41,42,43,44,45,46]. Given the low pathogen exposure threshold in chicks, our results highlight the brooding period as a critical window for intensified bioaerosol risk control during winter.

On the other hand, beneficial genera with significance for gut homeostasis, including *Bacteroides*, *Ruminococcus*, and *Lactobacillus*, became progressively dominant as the flock aged [47,48,49,50,51,52,53]. This suggests that as the chickens’ digestive systems matured, their gut microbiota more readily entered the air circulation and came to dominate the aerosolized community. This is not only a result of fecal aerosolization but may also reflect a long-term “ecological spillover” of gut flora into the indoor environment.

It is crucial to emphasize that some of the detected zoonotic-related genera are priority pathogens of national concern. This finding underscores the potential for occupational exposure and a One Health risk within these enclosed winter houses. Without proper environmental management and air monitoring, aerosol transmission could breach the confines of the poultry house, establishing a pathway for regional dissemination and thereby threatening surrounding public health. Consequently, managing airborne microorganisms in winter poultry operations should not be viewed solely through the lens of disease control. It must be integrated into a One Health framework and advanced in parallel with strategies for animal welfare, laborer protection, and environmental sustainability.

This study provides significant insights into the ecological succession patterns of bacterial aerosols in enclosed poultry houses during the critical winter season. Based on our findings, the following strategies are recommended for poultry farms in cold regions: Optimize winter ventilation systems to ensure essential air exchange while maintaining low energy consumption. Enhance disinfection protocols and implement rapid manure removal procedures, particularly during the brooding period. Equip farm workers with certified personal respiratory protective equipment. Establish routine airborne microbial monitoring systems for early detection of potential zoonotic pathogens. Future research should integrate pathogen genomics, investigations into antibiotic resistance gene dissemination, dynamic particulate matter modeling, and host respiratory immune responses. Such multidisciplinary approaches will provide a scientific foundation for developing intelligent, low-carbon, and biosecure management strategies for poultry farming environments.

## 5. Conclusions

This study systematically elucidates the dynamics of airborne bacterial aerosols in enclosed, cage-housed Taihang chicken facilities during winter, revealing pronounced age-dependent patterns in concentration, particle-size distribution, and community structure. Culturable airborne bacterial concentrations increased progressively across growth stages, accompanied by a notable shift from coarse particles to fine respirable fractions capable of deep lung deposition. This particle-size migration substantially heightens respiratory exposure risks for both poultry and farm workers under winter minimum-ventilation conditions. High-throughput sequencing further demonstrated increasing bacterial diversity with flock age, characterized by a clear successional transition from environmentally derived taxa toward host-associated genera.

Our findings highlight a dual ecological pattern in which potentially pathogenic and beneficial bacteria coexist within the airborne microecosystem. Zoonotic-related taxa were most abundant during the chick stage, reinforcing the brooding period as a critical window for bioaerosol exposure control. Conversely, beneficial genera associated with gut homeostasis became increasingly dominant as birds matured, reflecting the strong ecological coupling between host physiological development, fecal aerosolization processes, and airborne microbial dispersal. This successional trajectory underscores the intrinsic plasticity of the airborne microbiome within intensive poultry environments.

Overall, this study provides mechanistic insight into the formation pathways and ecological succession of bacterial aerosols in winter poultry houses. These findings offer a scientific basis for optimizing ventilation management, airborne contamination control, and biosecurity practices in Taihang chicken production systems in cold regions. From a One Health perspective, future strategies should integrate intelligent ventilation regulation, rapid manure and dust management, continuous airborne microbial monitoring, and strengthened occupational protection to mitigate aerosol transmission risks and promote animal welfare, human health, and environmental sustainability.

## Data Availability

All original contributions of this study are contained within the article; further details are available from the corresponding authors upon request.

## Supplementary Materials

The following supporting information can be downloaded at https://www.mdpi.com/article/10.3390/ani15243635/s1. Figure S1: Rarefaction curves illustrating the sequencing depth and OTU richness of airborne bacterial communities collected from Taihang chicken houses at three different growth stages (15 days, 30 days, and 150 days). Table S1: Relative abundance (%) of dominant bacterial genera in airborne bacterial communities at 15, 60, and 150 days of age.
