# Supplementary material for: Ecological Succession of Airborne Bacterial Aerosols in Poultry Houses: Insights from Taihang Chickens
Source: Animals (Basel). 2025 Dec 17;15(24):3635. doi: 10.3390/ani15243635 (PMC12729644; doi:10.3390/ani15243635)
Supplement: Supplementary file 1 [file animals-15-03635-s001.zip › animals-4021014-supplementary.pdf]

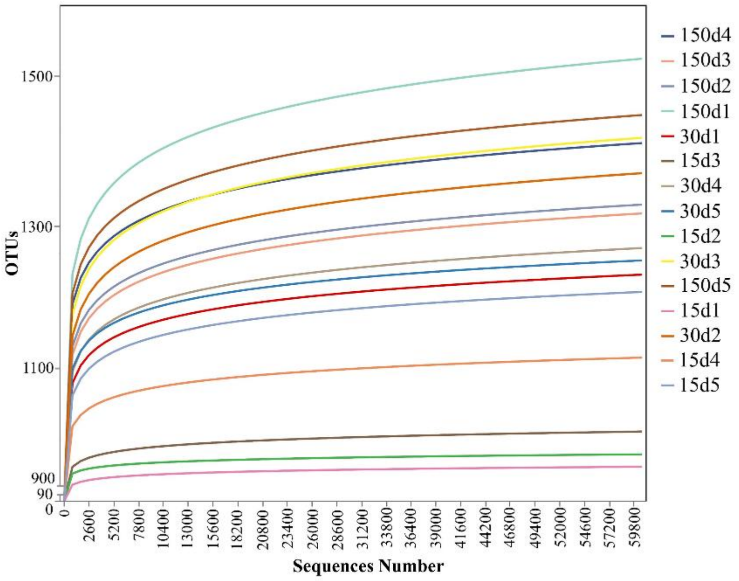

Supplementary Figure S1. Rarefaction curves illustrating the sequencing depth and OTU richness of airborne bacterial communities collected from Taihang chicken houses at three different growth stages (15 days, 30 days, and 150 days). Each curve represents an individual sample (n = 5 per stage). All curves approach a plateau, indicating that the sequencing depth was sufficient to capture the majority of bacterial diversity within each sample. Rarefaction analysis was performed using QIIME2 based on the 16S rRNA gene sequencing dataset.

Supplementary Table S1. Relative abundance (%) of dominant bacterial genera in airborne bacterial communities at 15, 60, and 150 days of age.

No.	Genus	15 d (%)	60 d (%)	150 d (%)
1	*Acinetobacter*	37.90%	21.68%	0.33%
2	*Bacteroides*	6.62%	24.48%	25.78%
3	*Faecalibacterium*	4.31%	6.23%	12.86%
4	*[Ruminococcus]_torques_group*	1.91%	7.45%	8.72%
5	*unidentified_Chloroplast*	8.11%	1.87%	5.25%
6	*Lactobacillus*	1.69%	4.54%	7.05%
7	*Aerococcus*	7.45%	3.77%	0.33%
8	*Corynebacterium*	8.11%	1.56%	1.03%
9	*Fusobacterium*	0.37%	3.44%	6.84%
10	*Megamonas*	1.78%	4.32%	3.84%
11	*Psychrobacter*	1.30%	7.68%	0.05%
12	*unidentified_Mitochondria*	1.26%	1.63%	6.30%
13	*Olsenella*	0.23%	2.50%	5.15%
14	*Methylobacterium-Methylorubrum*	0.55%	0.49%	6.27%
15	*Rikenellaceae_RC9_gut_group*	0.56%	2.34%	4.87%
16	*Sphingobacterium*	4.92%	2.10%	0.09%
17	*Ligilactobacillus*	1.24%	2.96%	3.72%
18	*pseudomonas*	5.02%	0.47%	0.17%
19	*Staphylococcus*	3.61%	0.49%	1.37%
20	*Luteimonas*	3.07%	0.02%	0.01%
